# Metabolism of Neonatal Vitamin A Supplementation: A Systematic Review

**DOI:** 10.1093/advances/nmaa137

**Published:** 2020-11-19

**Authors:** Bryan M Gannon, Lisa M Rogers, Sherry A Tanumihardjo

**Affiliations:** Interdepartmental Graduate Program in Nutritional Sciences, University of Wisconsin-Madison, Madison, WI, USA; Division of Nutritional Sciences, Cornell University, Ithaca, NY, USA; Department of Nutrition and Food Safety, WHO, Geneva, Switzerland; Interdepartmental Graduate Program in Nutritional Sciences, University of Wisconsin-Madison, Madison, WI, USA

**Keywords:** neonate, newborn, retinol, supplementation, vitamin A

## Abstract

A systematic review was conducted to summarize the absorption, transport, storage, and metabolism of oral neonatal vitamin A supplementation (NVAS). This review focused specifically on the neonatal period (first 28 d of life for humans) to inform guidance by WHO on recommendations related to NVAS. A systematic search of international and regional databases was conducted. Inclusion criteria were human or animal studies that gave oral vitamin A as a single or limited number of doses to apparently healthy neonates. Studies evaluating fortification or food-based approaches, dosing with retinoic acid, or studies of neonatal models of disease were excluded. The search retrieved 8847 unique records. After screening by title and abstract, 88 were screened using the full text, and 35 records met inclusion criteria: 13 human and 22 animal studies. Studies indicate that high-dose NVAS is absorbed well by neonates, typically mirroring fat absorption. Doses were primarily stored in the liver and transiently increased in the lung, kidney, spleen, adrenal glands, brain, skin, and adipose tissue, generally with a dose-response. Serum retinol and retinyl esters also transiently increased following NVAS. Although minimal acute adverse effects are noted, there is a lack of data supporting NVAS for improving organ maturation or sustained delivery to target organs. Research gaps include the physiological effects of the short-term increase of vitamin A concentrations in extrahepatic tissues, or whether there are unknown adverse effects over time.

## Teaser

Systematic review and summary of human and animal studies on the absorption, transport, storage, and metabolism of oral vitamin A supplementation given to newborns.

## Introduction

### Vitamin A and global public health relevance

Vitamin A (VA) is an essential nutrient in humans that has roles in normal immune function, cellular differentiation, embryogenesis, hematopoiesis, and the visual cycle (1). VA deficiency causes significant public health problems including blindness, decreased immune function, and death (2, 3). Infants, children aged <5 y, and pregnant and lactating women are especially vulnerable to deficiency due to their increased needs to support growth and exposure to nutrient-depleting infections (4). It is estimated that 29% of preschool-aged children in low- and middle-income countries (LMICs) are VA deficient (defined as a serum retinol concentration <0.7 μmol/L) (5) and 15% of pregnant women globally are VA deficient (serum retinol <0.7 μmol/L),  with the WHO regions of Africa and South-East Asia having the highest prevalence and greatest number of individuals affected (6). Although there have been improvements in maternal, child, and infant health in the recent past, these still remain a major global health concern (7).

Of the life stages within children under 5 (i.e. neonates aged <28 d, infants aged <1 y, and young children aged <5 y), neonates experience a disproportionate burden of deaths (8). Of the 6.6 million deaths among children under 5 y in 2012, 4.8 million were infant deaths, of which 2.9 million were neonatal deaths, indicating a high importance for this age group and also a high impact opportunity if successful interventions are found (9). Although the majority of neonatal deaths (deaths occurring within the first 28 d of life) are not thought to be directly related to VA deficiency (prematurity and low birth weight, 31%; birth asphyxia/trauma, 23%), infections and diarrheal diseases cause 25% and 2.6% of neonatal deaths, respectively, and improving VA status is hypothesized to reduce these important causes of death (10). VA plays important roles in innate and adaptive immunity. In animal and human studies, VA deficiency impaired various immune functions, however, repletion with retinyl ester, provitamin A carotenoid, or the active hormone, retinoic acid, restored functions (11–13). These factors may account for the increased mortality seen in VA-deficient infants and young children.

Newborns typically have low serum VA concentrations and low liver stores of VA at birth due to the highly regulated maternal transfer of VA to the fetus (14). Additionally, newborns in less developed countries may have lower VA stores (15). Colostrum and breast milk of well-nourished women are rich sources of VA. When a mother is VA deficient or not consistently consuming VA during the lactation period, her breast milk may have low VA, resulting in low VA intakes and preventing a build-up of hepatic VA reserves (16). Breast milk VA concentrations in unsupplemented mothers in LMICs are lower than counterparts in high-income countries. The average breast milk VA concentrations in LMICs are still enough to meet the needs of the infant to prevent clinical VA deficiency (e.g. eye signs), but do not allow for storage in the liver past 6 mo, leaving the infant susceptible to deficiency due to complementary feeding or postweaning diets low in VA and/or infection. Healthy, breastfed infants without signs of VA deficiency have intakes between 120 and 170 μg retinol activity equivalents (RAE)/d (400–567 IU/d) in some LMICs (17). Furthermore, differences in breast milk VA concentrations exist among LMICs; a multicenter trial showed a large difference in breast milk VA content in Ghana (59.9 ± 25.4 nmol/g fat) compared with India (38.1 ± 25.4 nmol/g fat) (*P* < 0.0001) (18).

Postpartum VA supplementation was previously recommended by WHO for the improvement of maternal VA status and infant VA status through increased breast milk VA concentrations (19). However, as of 2011, maternal VA supplementation is no longer recommended as a public health intervention based on lack of evidence for the prevention of maternal and infant morbidity and mortality (20).

### VA metabolism

VA can be obtained through the consumption of preformed VA, the predominant form in animal-source foods, fortification, and supplementation, or provitamin A carotenoids, primarily from plant-source foods. Dietary VA is incorporated into micelles in the intestine, absorbed by enterocytes, and packaged into chylomicra. These chylomicra are then transferred through the lymph into the bloodstream where they can deliver VA directly to target tissues.

Chylomicron remnants are then taken up by the liver, the primary VA storage site, where VA can be stored as retinyl ester or complexed as retinol:retinol-binding protein (RBP) as the primary form for circulation. In tissues, VA can then be converted to retinal for function in the visual cycle or to retinoic acid, the hormone form of VA that controls the expression of numerous genes and drives the differentiation of many cell types (21).

### VA supplementation

High-dose neonatal VA supplementation (NVAS) seeks to immediately supply the neonate with a large dose of VA that can be stored and used for months to prevent deficiency and associated morbidities if the dietary intake of the neonate is low in VA. A common dose evaluated for human neonates is 15,000 μg (50,000 IU). VA supplementation of children aged 6–59 mo reduced child mortality by 24% in a meta-analysis, mainly due to a reduction in deaths related to diarrhea and measles (22). However, there have been contrasting results of NVAS on infant mortality. Trials in Indonesia (23), India (24), and Bangladesh (25) demonstrated a reduction in infant mortality (15–64%), yet trials in Nepal (26), Zimbabwe (27), and Guinea-Bissau (28, 29) had no effect on infant mortality (8). Three additional trials were carried out in India (30), Ghana (31), and Tanzania (32) that demonstrated no impact on infant mortality, but in India there was a borderline significant reduction in mortality at 6 mo with NVAS (RR: 0.90, 95% CI: 0.81–1.00, *P* = 0.056). This has been a subject of considerable debate for WHO and global public health (8, 33, 34).

High-dose supplementation is generally given as preformed VA (retinyl palmitate). The estimated safe adequate intake of VA in infants aged <6 mo ranges from 375 μg RAE/d (35) to 400 μg RAE/d (36) (1250–1333 IU, respectively), based on average values of milk intake and breast milk VA concentrations in healthy populations. Recommendations for safe upper limits on daily VA intake come from applying uncertainty estimates to lowest-observed-adverse-effect levels, with estimates at 600 and 900 μg/d (2000–3000 IU/d) of preformed VA for children (35, 36). It is noted that NVAS exceeds this daily upper intake with a typical dose for human neonates being 15,000 μg (50,000 IU), and therefore consideration for the risk of toxicity is present.

Generally recognized symptoms of acute VA toxicity include anorexia, bulging fontanelle, drowsiness, increased intracranial pressure, irritability, diarrhea, and vomiting (37). Haider and Bhutta (38) found a significant increase in bulging fontanelle with NVAS and nonsignificant decreases in vomiting and diarrhea within 3 d of supplementation. Humphrey and Ichord (39) concluded that although there may be slight increases in these risks, they are uncommon, mild, and unlikely to discourage communities from supplementation.

Additionally, concerns have been raised about a possible negative interaction among NVAS, sex, and immunizations. Specifically, it is hypothesized that NVAS may lead to negative mortality outcomes for females once they start receiving the diphtheria, tetanus, and pertussis (DTP) vaccine (40), which could add to explanations regarding heterogeneity among NVAS trials (41).

While clinical trials have not confirmed the effectiveness of NVAS on infant mortality, the VA status of susceptible populations still needs to be addressed. Until optimal nutrition is achieved through other means, high-dose supplementation offers an interim solution, but little is known regarding physiological characteristics of the dose, the full extent of its actions, and the safety associated with it.

This review will help to identify potential mechanisms through which VA may impact infant survival, so that future studies may use appropriate methods to determine the most effective way to reduce infant morbidity and mortality through improving VA status and associated health benefits.

### Animal models of VA metabolism

Rats and swine are 2 appropriate and commonly used models to study VA deficiency and related mechanisms including absorption, transport, storage, and metabolism of doses (42). Mice and preruminant calves are also used to study VA metabolism, with mice commonly used for applications involving immune function or cancer and preruminant calves have been used to mimic human provitamin A carotenoid metabolism (42).

Rats are considered an appropriate model to study VA deficiency, immune function, and lactation due to similarities in organs, enzymes, transport proteins, and receptors involved in retinoid metabolism (42). Similar to humans, rats are born with low tissue concentrations of VA and obtain VA through milk, the VA content of which is dependent on the maternal diet (43). Rats have been used extensively in studies of VA concerning reproduction and embryonic development (44, 45). VA is essential in lung development (46), and the neonatal rat model is used to investigate mechanisms for VA uptake by the lungs (47–49). The rat model has been used for VA metabolite excretion, whole-body and organ-specific kinetics, and body stores with the use of isotopically labeled VA (50). At term birth, rats have a relatively immature gastrointestinal tract (GIT) compared with humans, but it matures quickly around weaning (18–22 d) (51).

Swine are similar to humans in terms of their biology, especially notable for renal physiology/morphology, digestive physiology/morphology (52, 53), and lipoprotein/cholesterol metabolism (54). The human GIT begins developing early in the fetus, maturing slowly and consistently throughout weaning. Swine have a relatively comparable GIT to humans, although at term birth swine have a slightly less developed GIT that develops faster from just before birth until weaning (51). Because of these similarities, the pig is a good model for human nutrition and has been used for gastrointestinal function, infant nutrition studies, carbohydrate/lipid/amino acid metabolism, and vitamin/mineral requirements (55, 56). Like humans, piglets are born with low liver reserves and obtain their VA from the milk of lactating sows (57), thus making them an appropriate model to study nutrition of the breastfeeding mother-child dyad. Young piglets have been used to study NVAS effects on VA status in the liver (58) and extrahepatic organs (59). VA analogs have also been used as tracers in the sow-piglet dyad (60) and in sows (61) to study VA kinetics. Notable differences from humans that should be taken into account are the relatively low fat stores of the newborn piglet and higher rates of metabolism and growth in early life (62).

### Rationale and objective

Although NVAS is currently not recommended by WHO (63) for the prevention of morbidity and mortality, there has been much interest in this intervention using a single dose of 50,000 IU given within the neonatal period (38, 64–66), particularly within the first few days of life (8, 67). Questions remain on possible reduction in mortality at 6 mo in some settings, but importance is currently placed on safety and full understanding of the intervention before further recommendations can be made. This systematic review was conducted to evaluate the metabolism of NVAS to understand potential mechanisms of action. Our primary review question was: what is the absorption, metabolism, and storage of oral VA given to apparently healthy neonates? The animal models evaluated provide unique insights into the mechanisms through which VA can impact the neonate and have effects on infant mortality.

## Methods

### Protocol

An internal protocol was developed by the study authors prior to conducting this systematic review.

### Article search

The search was conducted in consultation with a WHO librarian to include human and animal studies without language restriction. Primary and secondary searches were conducted: primary, MEDLINE via PubMed, CINAHL, CAB direct, Web of Science, Agricola, Agris, WHO regional databases, clinical trial registries, and gray literature with no beginning date restrictions up until 20 August, 2013 and a secondary search of MEDLINE via PubMed between 20 August, 2013 and 5 January, 2020 using the following search terms: (newborn OR infant OR infants OR infancy OR neonat*) AND (“vitamin A” OR retinol OR retinyl OR retinoic). Additional searches were conducted using forward and backward citations of identified articles and other relevant review articles.

### Trial selection

The following inclusion criteria were required: *1*) apparently healthy human or animal neonates, defined as 0–28 d of life for newborn humans, 0–14 d for rats and mice, 0–21 d for swine, and 0–28 d for calves; *2*) given oral VA as a single dose or limited number of doses (i.e. not daily supplementation), provided as retinyl palmitate or retinyl acetate, or one of its analogs as tracers (e.g. isotopically labeled retinol [^2^H, ^3^H, ^13^C, ^14^C], vitamin A_2_ [3,4-didehydroretinyl acetate], α-retinol) during the species-specific neonatal period. The VA supplement may have been given alone or in combination with other micronutrients; however, studies were included if the only difference between treatment groups was VA, or if studies specifically evaluated VA tracer or kinetic outcomes. Studies directly supplementing with the hormone form of VA, retinoic acid, were excluded. If studies included multiple treatment groups, only groups that followed the inclusion criteria were considered.

Studies evaluating tube feeding, parental nutrition, or supplementary food-based interventions, such as food fortification, home fortification with micronutrient powders, lipid-based supplements, biofortification, the consumption of VA-rich foods, or β-carotene supplementation were not included. Studies focusing specifically on neonatal models of disease (e.g. bronchopulmonary dysplasia, cystic fibrosis, HIV) were not included in this review. These studies are of clinical importance but do not address the global public health research question of this review. If studies provided additional interventions, they were included if the only difference between the treatment arms was VA supplementation.

### Outcomes

All outcomes were assessed if measured between the time after supplementation and 6 mo of age for humans; animal studies did not prespecify a maximum follow-up length. The primary outcomes were categorized to correspond with specific areas of VA metabolism: *1*) VA absorption: short-term serum retinol and retinyl ester response and unabsorbed dose collected in feces; *2*) VA transport: long-term serum retinol and retinyl ester response, short-term organ retinol and retinyl ester concentrations, VA analog/tracer distribution; *3*) VA storage: long-term organ VA concentrations, biochemical markers of VA status over time; *4*) VA metabolism/detoxification: VA homeostatic gene expression, polar metabolite quantification; and *5*) organ maturation: weights, histology, biological assay. Secondary outcomes included any adverse effects observed. Outcomes related specifically to immune function will be analyzed and reported separately.

### Data management and analysis

Review authors assessed the potential eligibility of the identified references by conducting relevance screening. One review author (BMG) assessed all references beginning with the titles and then abstracts, if they were available. If a decision could not be made by screening the title and the abstract, the full text of the article was retrieved and reviewed. The full text of all articles that could potentially meet the inclusion criteria were retrieved and independently assessed for study eligibility using forms designed in accordance with the specified inclusion criteria in coordination with another author (LMR). A third review author (SAT) was consulted in the event of any disagreements.

For all eligible studies, data were extracted by 1 review author (BMG) using a form designed for this review and a second review author (LMR) carried out checks for accuracy. Disparities were discussed with the third review author (SAT). Data collection was done electronically, and detailed information was recorded (**Supplemental Figure 1**).

## Results

### Search results

The primary search resulted in a total of 8847 unique records from all databases after duplicates were removed, from which 35 records were ultimately included for outcomes; reasons for exclusion are outlined (**Supplemental Figure 2**). Thirteen of the records were conducted in humans; 7 in the USA, 2 in Zimbabwe, 2 in Guinea-Bissau, 1 in Indonesia, and 1 in Nepal. Twenty-two of the records were in other animal models: 11 in rats, 1 in mice, 6 in swine, and 4 in calves.

### Primary outcome measures

#### VA absorption

##### Short-term serum retinol response

Short-term (0–24 h postdose) serum retinol response was first assessed in the late 1940s in human and calf models to determine the absorption of different dosing preparations (retinyl ester versus retinol, oily versus aqueous preparation). More recently, it has been used to assess absorption, possible toxicity, and dose distribution in rats, swine, and humans (**Table 1**).

After NVAS, retinol and retinyl esters rose 3–9 fold within 3–6 h (68–72) and decreased by 24 h (70–72) in humans. Similar results were noted in calves, with 1 report seeing a similar fold increase by 4 h (73) and another seeing an increase by 12 h (first postdose measurement) (74). Piglets given NVAS had higher serum retinol and retinyl ester concentrations than control piglets within 24 h of dosing but not thereafter, and retinyl esters showed a dose response with amount dosed through 24 h (75, 76). Calves given 25,000 IU VA daily and skim milk maintained prefeeding plasma VA concentrations similarly to calves given colostrum and whole milk (∼0.38 μmol/L), whereas those given only 10,000 IU VA with skim milk tended to have lower serum VA (∼0.15 μmol/L) (77). Rats receiving NVAS on postnatal days 5–7 had higher plasma retinol than controls 24 h after the last dose: (∼1.55 compared with ∼1.05 μmol/L, respectively, *P* < 0.05) (78) and (1.8 compared with 1.4 μmol/L, respectively, no statistics reported) (79). Rats dosed on postnatal day 4 had elevated serum retinol and retinyl ester concentrations compared with controls peaking at 1 h and returning to control concentrations by 8–15 h after dosing, with the increase primarily representing retinyl esters associated with chylomicra (80, 81). Full-term human neonates tended to have a higher serum retinol response than prematurely born neonates (69), and aqueous preparations (solubilized in propylene glycol, glycerin, or another dispersing agent) had a significantly higher serum retinol response than the oily preparation (68, 70, 71).

##### Unabsorbed dose collected in feces

Assessing apparent absorption by collecting the unabsorbed dose in feces was done in the early 1950s in a few human studies, a calf study, and a recent swine study (Table 1). Absorption, calculated by subtracting fecal excretion, was 78% (*n* = 1) (70), 70 ± 18% (*n* = 8) (82), 44 ± 18% (*n* = 5) (83), and 61 ± 9% (*n* = 14) (84). Absorption was independent of fat intake (82) and generally mirrored fat absorption (82–84); emulsification of the dose, which reduces the particle size, increased absorption by ∼20% (83). Calves showed minimal fecal excretion, but excretion was higher in calves with diarrhea (73). Colon contents of piglets contained variable and substantial (5–25% of dose) VA 48 h after dose administration (76).

**TABLE 1:** Characteristics of included studies evaluating vitamin A absorption in neonates

Model	Dose amount (*n*/group)	Frequency, age at dosing	Outcome	Reference
			***Short-term (0–24 h) serum/plasma vitamin A response***	
Calf	10,000 IU (3)25,000 IU (13)Control (colostrum) (4)	Multiple, days 1–14	25,000 IU group similar plasma VA increase as colostrum group. 10,000 IU group increased to ∼50% the concentration of other groups^1^	(Hansen, 1946) (77)
Calf	250,000 IU (3)Control (colostrum) (3)	Single, day 1	250,000 IU group plasma VA increased significantly to ∼3 fold higher than control group	(Eaton, 1947) (74)
Calf	59,000–500,000 IU (17)	Single, day 1	Healthy calves had ∼3–5-fold increase in plasma VA; minimal increase in scouring calves^1^	(Sellers, 1949) (73)
Human	17,000 IU/kg (15)	Single, day 6–28	Mature neonates had ∼3-fold greater plasma VA increase than premature infants^1^	(Henley, 1944) (69)
Human	35,000 IU (2)	Single, ∼day 28	Aqueous preparation resulted in ∼5-fold greater plasma VA increase than oily preparation^1^	(Lewis, 1947) (70)
Human	35,000 IU oily (31)16,000–24,000 IU aqueous (33)	Single, day 3–28	Aqueous preparation resulted in ∼5-fold greater plasma VA increase than oily preparation^1^	(Clifford, 1948) (68)
Human	13,500 IU/kg (39; 14 of which had aqueous and oily preparations, 7 of which had ester and alcohol forms in oil)	Single (double for comparisons), “newborn”	Aqueous preparation resulted in ∼3-fold greater serum VA increase than oily preparation^1^Ester and alcohol forms in oil had similar serum VA responses	(Sobel, 1949) (71)
Human	50,000 IU (9)	Single, day 1–28	Serum retinol and retinyl esters rose at 6 h and reduced at 24 h following dosing	(Agoestina, 1994) (72)
Rat	6 μg/g (50,000 IU to humans) (4)Control (4)	Multiple, days 5–7	VA group mean plasma retinol estimate 1.3-fold higher than control group^1^	(Ross, 2006a) (79)
Rat	6 μg/g (50,000 IU to humans) (4)Control (4)	Multiple, days 5–7 or single, day 6 or 7	VA group 1.3-fold higher plasma retinol than control group	(Wu, 2010) (78)
Rat	6 μg/g (50,000 IU to humans) (24)Control (24)	Single, day 4	VA group has higher serum retinol and retinyl esters peaking at 1 h and returning to control levels by 8–15 h	(Hodges, 2016a; Hodges, 2017) (80, 81)
Swine	25,000 IU (20)50,000 IU (16)Control (10)	Single, day 1	VA groups had higher serum retinol and retinyl ester concentrations than control piglets with a dose response	(Heying, 2015) (75)
Swine	25,000 IU (83)50,000 IU (73)200,000 IU (71)Control (58)	Single, day 1	VA groups had higher serum retinol and retinyl ester concentrations than control piglets with a dose response	(Gannon, 2017) (76)
			***Unabsorbed dose collected in feces or colon contents***	
Calf	59,000–500,000 IU (17)	Single, day 1	Higher unabsorbed VA in feces of scouring calves^1^	(Sellers, 1949) (73)
Human	35,000 IU^2^ (1)	Single, ∼day 28	Absorption estimate: 78%	(Lewis, 1947) (70)
Human	2500–25,000 IU^2^ (8)	Multiple, days 11–28	Absorption estimate: 70 ± 18%	(Morales, 1950a) (82)
Human	28,000 to 57,000 IU^2^ (5)	Multiple, days 6–28	Absorption estimate: 44 ± 18%	(Morales, 1950b) (83)
Human	20,000 to 22,000 IU^2^ (14)	Multiple, days 4–28	Absorption estimate: ∼60%	(Snyderman, 1953) (84)
Swine	25,000 IU (3)50,000 IU (3)200,000 IU (3)Control (3)	Single, day 1	Colon contents of piglets contained variable and substantial (5–25% of dose) VA 48 h after dose administration	(Gannon, 2017) (76)

1 Statistics not specified for comparison of interest.

2 Specified in text as ‘units’; IU assumed based on methods reported; VA, vitamin A

##### Summary

High-dose VA is absorbed and appears in the serum as retinyl esters and retinol. VA absorption generally mirrored fat absorption, with estimates ranging from 44 to 78%, with considerable variation among studies. Absorption was lower in premature neonates and higher with reduced particle size following emulsification.

#### VA transport

##### Long-term serum retinol response

Long-term (>24 h postdose) serum retinol response was assessed in calf studies in the late 1940s and in recent studies in rats and piglets (**Table 2**). Calves given large VA doses at birth generally had higher serum VA concentrations than controls from 24 h to 42 d after birth, although similar values were noted at 21 d (74). Calves given a large dose on day 3 and day 10 of life had higher serum VA than controls on 10 and 20 d of life (85). Rats given single (80, 81) or multiple (86) high doses of VA did not have different plasma VA concentrations than controls by 15 h or 2 d postdosing, respectively. Control rats had higher total plasma retinol mass at 14 d but no other times before or after; total retinol concentration and retinyl ester concentration was similar between groups at 2–24 d (80). Piglets given single doses of VA had higher serum retinol concentrations 1 d postdose, but serum retinol was similar to controls on day 2 through to 10 postdose (58, 76).

##### Short-term (≤2 d) organ VA concentrations

Short-term organ uptake of VA was primarily assessed in rats, but also included swine, mouse, and calf studies (Table 2). The liver is the predominant site of VA clearance. Liver concentrations showed a dose response, accumulated 7–55% of the dose, and demonstrated elevated concentrations relative to control groups (47, 49, 73, 75, 76, 78–81, 86–88). The lung displayed a moderate increase in VA, increasing ∼2–5-fold above control concentrations (47, 49, 75, 76, 78–81, 88, 89). Fluorescence microscopy revealed that the VA dose was most concentrated in oil globules in the liver and adrenal glands in rats (87). Piglets had large relative increases in lung, kidney, spleen, and adrenal gland VA concentration by 7 through to 24 h, and displayed a dose response to the amount of VA given (75, 76). Rats also had higher VA concentrations in the stomach for 1 h; skin for 15 h; intestine, lung, and brain for 24 h; kidney for 48 h; and liver, brown adipose tissue, and white adipose tissue for >48 h (80, 90). Healthy birth weight piglets (>1.5 kg) had similar kidney and liver VA concentrations, but significantly greater total liver and kidney VA, when compared with low birth weight piglets (<1.0 kg), explained in part by increased organ size (75).

##### VA analog/tracer distribution

VA analog or tracer distribution in a neonatal model has only been studied within the past 10 y, using rat or swine models (Table 2). Labeled VA ([^3^H]-retinol) was given to rats as a tracer with high doses or placebo to assess VA uptake. Rat liver took up a higher percentage of the tracer when given high-dose VA compared with control; however, fractional uptake by the lung was similar between the VA and placebo groups (78, 79). The fraction of tracer in ester form was increased slightly with VA treatment (79). NVAS altered numerous kinetic parameters related to VA metabolism in the brain including increased fractional uptake and subsequent loss of chylomicron retinyl esters, and decreased fractional uptake and subsequent loss of retinol bound to RBP (90). A further report indicated that tracer in serum peaked between 1 and 4 h postdosing, and the liver was the predominant VA storage site in rats receiving NVAS as well as in control rats. Compartmental modeling of these tracer data demonstrated that NVAS redirected chylomicron retinyl esters away from the skin and carcass and towards the liver for greater fractional release as retinol bound to RBP (91). The amount of VA turnover and VA recycling between organs and plasma was greater in VA-treated rats (91).

Following NVAS, the tracer concentration in plasma was significantly higher at 1 h and significantly lower at 4 and 24 h following dosing compared with placebo (81). Tracer concentration in the liver was significantly higher with NVAS compared with placebo at 4 to 15 h following dosing, did not differ between 1 and 4 d, and was again significantly higher 8–14 d postdosing. Tracer concentration was significantly elevated in the lung 11–14 d, and significantly lower in the kidney 8–24 h postdosing with NVAS. There was no difference in tracer response in the stomach, intestine, or carcass between NVAS and control animals. Compared with placebo, NVAS increased the fractional release of VA from the liver, increased recycling of VA from organs to plasma, lowered the fractional loss of VA from organs, and affected other related kinetic parameters of VA metabolism. The uptake and turnover of modeled RBP-retinol and chylomicron-retinyl esters were altered in the stomach, intestine, liver, lung, kidney, and carcass with NVAS (81).

Labeled VA ([^13^C]-retinol) given to piglets followed a similar serum:liver distribution pattern seen in studies of older humans or animals, first appearing in the serum at 1–2 d following dosing, remaining higher in serum than liver at day 4, decreasing at day 7, and trending lower at 14 d with a 0.91 serum:liver enrichment ratio, indicating the dose mixed with the body pool and had lower enrichment in serum than the liver due to a small amount of VA coming through the diet (92).

Vitamin A_2_ (3,4-didehydroretinyl acetate; PubChem CID: 6,436,043), an analog of retinol with an extra double bond at the 3,4 position, was given to piglets 10 d after high-dose VA or placebo to assess VA uptake following treatment (59). The dose predominantly went to the liver (16–51%), but also to the spleen, lung, adrenal gland, and kidney. Relatively high values of vitamin A_2_ at 4 h after A_2_ dosing indicated that a significant amount of the dose was delivered by chylomicra. VA treatment and maternal VA status correlated with vitamin A_2_ found in the liver, but not extrahepatic organs.

**TABLE 2:** Characteristics of included studies evaluating vitamin A transport in neonates

Model	Dose amount (*n*/group)	Frequency and age at dosing	Outcome	Reference
			***Longer-term (>24 h) serum/plasma retinol response***	
Calf	250,000 IU (9)Control (9)	Single, day 1	Calves given large VA doses at birth generally had higher concentrations than controls from 24 h to 42 d after birth, although values were not different at 21 d	(Eaton, 1947) (74)
Calf	250,000 IU (13)Control (10)	Multiple, days 3,10	VA group higher than controls on 10 and 20 d of life	(Hibbs, 1947) (85)
Rat	37.5 μg RAE (25,000 IU to humans) (3)150 μg RAE (100,000 IU to humans) (11)Control (11)	Single, day 8, ∼12, or 15	Similar among groups 2 d postdosing	(Gardner, 1995) (86)
Rat	6 μg/g (50,000 IU to humans) (28)Control (28)	Single, day 4	VA and control groups total retinol and retinyl ester concentrations similar by 15 h through to 24 d	(Hodges, 2016a; Hodges, 2017) (80, 81)
Swine	25,000 IU (16)50,000 IU (16)100,000 IU (16)Control (16)	Single, day 18	Similar among groups 10 d postdosing	(Surles, 2007) (58)
Swine	25,000 IU (83)50,000 IU (73)200,000 IU (71)Control (58)	Single, day 1	VA group higher with dose response at 1 d, but similar among all groups 4–10 d postdosing	(Gannon, 2017) (76)
			* **Short-term (≤2 d) organ vitamin A uptake***	
Calf	59,000–1,000,000 IU (17)	Single (most days 1–4, some days 12–21)	Recovery of VA dose ∼33% in liver of healthy calves; ∼11% in scouring calves	(Sellers, 1949) (73)
Mouse	6 μg/g (50,000 IU to humans) (6)Control (9)	Multiple, days 1–4	VA group higher liver and lung VA concentrations on day 4 of life	(James, 2010) (49)
Rat	25,000 IU (2)Control (8)	Single, day 5, 7, or 17	Fluorescence microscopy revealed that the VA dose was most concentrated in oil globules in the liver and adrenal glands in rats 1 d postdosing	(Radice, 1955) (87)
Rat	37.5 μg RAE (25,000 IU to humans) (3)150 μg RAE (100,000 IU to humans) (11)Control (11)	Single, day 8, ∼12, or 15	VA groups higher liver VA concentrations 2-d postdosing	(Gardner, 1995) (86)
Rat	6 μg/g (50,000 IU to humans) (4)Control (4)	Multiple, days 5–7	VA group higher liver and lung VA concentrations on postnatal day 8	(Ross, 2006a) (79)
Rat	6 μg/g (50,000 IU to humans) (4)Control (4)	Multiple, days 5–7	VA group higher liver and lung VA concentrations on postnatal day 8	(Ross, 2006b) (47)
Rat	6 μg/g (50,000 IU to humans) (4)Control (4)	Multiple, days 1–3	VA group higher lung VA concentration on postnatal day 4	(Ross, 2007) (89)
Rat	6 μg/g (50,000 IU to humans) (12–16)Control (12–16)	Multiple (days 5–7) or single (day 6 or 7)	VA group higher lung VA concentration at 6- and 12-h postdose	(Wu, 2010) (78)
Rat	6 μg/g (50,000 IU to humans)	Single (day 4 or 14) or multiple (days 4, 7, 11, 14)	VA group higher liver and lung VA concentrations on postnatal day 8	(Wu, 2013) (88)
Rat	6 μg/g (50,000 IU to humans) (28)Control (28)	Single, day 4	VA group higher VA concentrations in the stomach for 1 h; skin for 15 h; intestine, lung, and brain for 24 h; kidney for 48 h; and liver, brown adipose tissue, and white adipose tissue for >48-h postdose	(Hodges, 2016a; Hodges, 2016b; Hodges, 2017) (80, 81, 90)
Swine	25,000 IU (20)50,000 IU (16)Control (10)	Single, day 1	Liver, kidney, adrenal, and spleen higher in VA dosed groups at 12- and 24-h postdosingLung not significantly different between groups at 12- and 24-h postdosing	(Heying, 2015) (75)
Swine	25,000 IU (83)50,000 IU (73)200,000 IU (71)Control (58)	Single, day 1	Liver, lung, kidney, adrenal, and spleen had a dose response at 7–24-h postdosing	(Gannon, 2017) (76)
			***Vitamin A analog tracer distribution***	
Rat	6 μg/g VA (50,000 IU to humans) with 2 μCi [^3^H] retinol (day 10 only) (3)Control with 2 μCi [^3^H] retinol (day 10 only) (3)	Multiple, days 9, 10	Fractional uptake by lung similar between VA and control groups, but the fraction of tracer in ester form was increased slightly with VA treatment	(Ross, 2006a) (79)
Rat	6 μg/g VA (50,000 IU to humans) with 2 μCi [^3^H] retinol (4)Control with 2 μCi [^3^H] retinol (4)	Single (day 6 or 7)	Liver took up a higher percentage of the tracer in VAFractional uptake by lung was similar between groups	(Wu, 2010) (78)
Swine	Vitamin A_2_, 5.3 μmol, after 25,000 IU (7)50,000 IU (7)100,000 IU (7)Control (7)	VA day 18 vitamin A_2_ day 28	The dose predominantly went to the liver, but also to spleen, lung, adrenal gland, and kidneyLiver, but not extrahepatic organ, vitamin A_2_ correlated with VA dosing and maternal VA statusNormalized serum α-retinyl esters were not detectible at baseline and peaked between 4- and 10-h postdosing	(Sun, 2008) (59)
Swine	α-Retinol 25,000 IU (24)α-Retinol 50,000 IU (24)Control (6)	Single (day 1)	Kidney, lung, spleen, adrenal, and intestinal mucosa α-retinol concentrations showed a dose response, peaking between 7 and 24 h, and were not detectible or were extremely low by 7-d postdosingLiver α-retinol concentrations showed a dose response and maintained maximal concentrations through 7-d postdosing	(Riabroy, 2014) (93)
Rat	6 μg/g VA (50,000 IU to humans) with 1.8 μCi [^3^H] retinol (52)Control with 1.8 μCi [^3^H] retinol (52)	Single, day 4	Tracer response evaluated in plasma, liver, lung, kidney, stomach, intestine, carcass, brain, adipose from 0- to 24-d postdoseThe effect of VA supplementation on VA content in most extrahepatic organs is transient (*see description in text*)	(Hodges, 2017, Hodges, 2016b, Hodges, 2017b) (81, 90, 91)
Swine	1.75 μmol ^13^C_2_-retinyl acetate (24)	Single, day 17	Kinetics of labeled VA in serum, liver, and serum:liver ratio through 14 d postdose	(Sheftel, 2019) (92)

REA, retinol activity equivalents; VA, vitamin A.

α-Retinol (PubChem CID: 6,441,065 for α-retinyl acetate) is a structural isomer of retinol where the double bond in the ring portion of the molecule is shifted from the 5,6 position in retinol to the 4,5 position in α-retinol, which does not allow it to bind to RBP (94). Large doses of α-retinyl esters were given to newborn piglets (aged <1 d) to track chylomicron delivery of VA, and blood and organ concentrations of α-retinol and α-retinyl esters were assessed through 7 d postdosing (93). Normalized serum α-retinyl esters were not detectible at baseline and peaked between 4 and 10 h. Kidney, lung, spleen, adrenal, and intestinal mucosa α-retinol concentrations showed a dose response, peaked between 7 and 24 h, and were not detectible or were extremely low by 7 d. Liver α-retinol concentrations showed a dose response and maintained maximal concentrations through 7 d (93).

##### Summary

Neonates transport high-dose VA primarily to storage in the liver, but also to the lung, kidney, spleen, adrenal gland, brain, skin, and adipose tissue. VA status and VA supplementation amount correlated with dose uptake in the liver, but not extrahepatic tissues, likely due to limited capacity for long-term storage and preferential uptake from chylomicra instead of RBP. Healthy versus low birth weight status resulted in higher total VA in the liver and kidney. Earlier results suggest a sustained increase in serum retinol concentrations; however, more recent studies indicate that serum retinol is similar between dosed and placebo groups within 2 d postdosing.

#### VA storage

##### Long-term (>2 d) organ VA concentrations

Long-term concentrations of VA in organs were assessed in calf studies as well as more recent studies in rats and swine (**Table 3**). Liver VA was maintained at higher concentrations in dosed animals than controls at 7–11 d postdose in calves (74, 85), through to 11 and 14 d in rats (80, 81), and showed a dose response from 24 h through to 10 d postdose in piglets (58, 76). Furthermore, piglet liver storage was also dependent on the mother's VA status, with more of the dose stored in piglets from less deficient mothers; however, sow milk VA concentration did not differ between sow groups of differing VA status (58).

In piglets sampled 10 d postdosing, kidney and adrenal gland VA concentrations were higher in dosed piglets than controls 10 d after dosing (59). In a group receiving 50,000 IU VA, total spleen VA content was higher than controls, but spleen VA concentration was similar to controls, and lung VA values were similar among controls and all dosed groups. No dose response was observed in extrahepatic organs (59). Another piglet study demonstrated treatment differences in adrenal VA concentrations between 1 and 7 d postdosing, but not at 10 d, and treatment differences in lung, kidney, and spleen VA concentrations only at 1 d postdosing (76).

Lung VA concentrations were assessed 1 and 5 d postdosing in rats. VA-dosed groups maintained higher lung concentrations of VA over controls; however, lung VA concentrations in dosed groups decreased ∼50% between day 1 and 5 postdose, indicating rapid utilization or metabolism of the VA dose (89). Another rat study demonstrated that carcass, stomach, skin, intestine, lung, brain, and kidney VA concentrations did not differ between treated and control groups past 2 d; however, brown and white adipose tissue had significant differences through 4 and 18 d, respectively (80, 90). Different analysis with additional controls indicated that lung VA remained elevated relative to controls 8–14 d post NVAS (81).

##### Additional biochemical markers of VA status over time

RBP was assessed in 1 human study, and the modified relative dose response (MRDR) was assessed in 1 swine study in the mid 2000s (Table 3). The MRDR assesses VA status by dosing with vitamin A_2_ and determining the ratio of vitamin A_2_:VA in the alcohol forms ∼5 h following dosing (95).

NVAS did not alter RBP, which was used as a surrogate for serum retinol concentrations, at 6 wk (0.93 μmol/L) or 4 mo (1.06 μmol/L) of age (96). The MRDR values showed no difference in piglets from less VA-deficient mothers (all groups had mean MRDR values <0.06 indicating adequate VA status). MRDR values showed a treatment effect in response to graded doses of NVAS in piglets from more VA-deficient mothers (all groups had mean MRDR values >0.06 indicating deficient VA status) (58).

##### Summary

The liver is the primary storage site of VA in neonates, similar to older animals. When given varying concentrations of high-dose VA, the liver shows a dose response in storage at least through to 11 d after dosing. It is not known how long these stores that are acquired during the neonatal period remain elevated either through supplementation or breast milk. Extrahepatic tissues do not appreciably store high amounts of VA, with the kidney perhaps being an exception, especially during VA deficiency. Adipose tissue maintained higher VA concentrations in response to NVAS than some other organs; however, this still represents a much smaller pool than what is stored in the liver. The lung acquires VA from early chylomicron circulation, but concentrations are quickly reduced through utilization, metabolism, or excretion. Human neonates did not show a serum response in RBP concentrations 4 wk postdosing. These data indicate that NVAS is stored predominantly in the liver, but it is not known how long liver stores are maintained or how efficiently these stores are able to be utilized by other tissues.

**TABLE 3:** Characteristics of included studies evaluating vitamin A storage in neonates

Model	Dose amount (*n*/group)	Frequency and age at dosing	Outcomes	Reference
			***Longer-term (>2 d) organ vitamin A concentrations***	
Calf	250,000 IU (3)Control (3)	Single, day 1	Liver VA higher in VA group on postnatal day 3.5	(Eaton, 1947) (74)
Calf	250,000 IU (13)Control (10)	Multiple, days 3, 10	Liver VA 10-fold higher at day 21 in VA groups	(Hibbs, 1947) (85)
Rat	6 μg/g (50,000 IU to humans) (3)Control (3)	Multiple, days 1–3	Lung VA higher in VA group on postnatal day 8	(Ross, 2007) (89)
Rat	6 μg/g (50,000 IU to humans) (24)Control (24)	Single, day 4	Liver VA higher in VA group at 11 and 14 dCarcass, stomach, skin, intestine, lung, brain, and kidney VA not different between groups past 2-d postdosingDifferent analysis with additional controls indicated that lung VA remained elevated relative to controls 8–14-d postdoseBrown and white adipose VA higher in VA group through 4 and 18 d, respectively	(Hodges, 2016a; Hodges, 2016b; Hodges, 2017) (80, 81, 90)
Swine	25,000 IU (16)50,000 IU (16)100,000 IU (16)Control (16)	Single, day 18	Liver VA showed dose response at 10-d postdose	(Surles, 2007) (58)
Swine	25,000 IU (7)50,000 IU (7)100,000 IU (7)Control (7)	Single, day 18	Kidney and adrenal gland VA concentration and spleen total VA higher in VA groups at 10-d postdosing; dose response not observedLung not different between groups at 10-d postdosing	(Sun, 2008) (59)
Swine	25,000 IU (83)50,000 IU (73)200,000 IU (71)Control (58)	Single, day 1	Liver VA showed dose response from 4 through to 10-d postdoseAdrenal VA showed dose response at 4 and 7 d postdoseLung, kidney, and spleen VA was not different between groups at 4-, 7-, and 10-d postdose	(Gannon, 2017) (76)
			***Biochemical markers of vitamin A status over time***	
Human	50,000 IU (186, 292 at 6 wk, 4 mo)Control (183, 320 at 6 wk, 4 mo)	Single, <day 10	Retinol-binding protein not different between groups at 6 wk or 4 mo of age	(Fisker, 2007) (96)
Swine	25,000 IU (16)50,000 IU (16)100,000 IU (16)Control (16)	Single, day 18	Modified relative dose-response values indicated increased VA stores in VA treated piglets from deficient mothers, but not from adequate mothers	(Surles, 2007) (58)

VA, vitamin A.

#### VA metabolism/detoxification

##### VA homeostatic gene expression (e.g. *Lrat, Stra6, Cyp21a1, Cyp26b1*)

VA homeostatic gene expression was assessed in 2 rat studies in the early 2010s (**Table 4**). The first study gave NVAS on postnatal days 5–7, and saw increased expression of lung *Lrat* and *Cyp26b1*, but no change in lung *Stra6* or *Cyp26a1*, at 6 and 12 h following the last dose (78). The second study observed no effect on *Stra6, Lrat*, or *Cyp26b1* expression in the lung following NVAS on postnatal days 4 or 14. However, expression of *Stra6, Lrat*, and *Cyp26b1* in the lung was increased following multiple doses of NVAS (88).

**TABLE 4:** Characteristics of included studies evaluating vitamin A metabolism/detoxification in neonates

Model	Dose amount (*n*/group)	Frequency and age at dosing	Outcome	Reference
			***Vitamin A homeostatic gene expression***	
Rat	6 μg/g (50,000 IU to humans) (4–6)Control (4–6)	Multiple, days 5–7	*Stra6*: –*Lrat*: ↑*Cyp26a1*: –*Cyp26b1*: ↑6, 12 h after last dose	(Wu, 2010) (78)
Rat	6 μg/g (50,000 IU to humans) (4–6)Control (4–6)	Single (day 4 or 14) or multiple (days 4, 7, 11, 14)	*Stra6*: –, ↑*Lrat*: –, ↑*Cyp26b1*: –, ↑6 h after single or last multiple dose, respectively	(Wu, 2013) (88)
			***Polar metabolite quantification***	
Rat	6 μg/g (50,000 IU to humans) (3)Control (3)	Multiple, days 5–7	Low polar metabolite accumulation in lung (∼0.02% of dose)	(Ross, 2006a) (79)

↑, increased with NVAS; –, not different with NVAS.

##### Polar metabolite quantification

Polar metabolite quantification of a VA dose was done in a single rat study in 2006 using [^3^H]-labeled VA (Table 4). Very little (∼0.02% of the oral dose) was recovered in the aqueous fraction of lung extracts, indicating either there was low metabolism to aqueous products in the lung, or that aqueous byproducts do not accumulate in the lung (79).

##### Summary

VA treatment can increase homeostatic gene mRNA expression in neonatal lungs; however, variability exists that could be explained by NVAS dose time and frequency. In these studies, stable analogs of retinoic acid had more profound effects on gene expression, implying that conversion of VA to retinoic acid and retinoic acid catabolism is tightly regulated in the lung, as lung retinol/retinyl esters are increased with VA treatment. Furthermore, VA polar metabolites are not significantly produced in or stored by the lung.

#### VA effects on organ maturation

Organ maturation by weight and histology and hemoglobin was examined in calf, swine, rat, mouse, and human models.

##### Organ weights and hemoglobin concentration

Organ weights or hemoglobin concentration were assessed in 1 study each in calves, humans, and rats, and 3 in piglets (**Table 5**). Hemoglobin concentrations and prevalence of anemia (hemoglobin <105 g/L) were not affected by VA supplementation when compared with controls in calves (74) or placebo in humans (97). Piglet organ and relative organ weights (liver [58, 76] and extrahepatic [59, 76] including the kidney, lung, spleen, and adrenal gland) 10 d postdose and rat lung and liver relative weights after 3 doses on postnatal days 5–7 (79) were not affected by VA treatment compared with controls. Rat brain, stomach, intestine, liver, lung, kidney, brown adipose, white adipose, skin, and carcass weights were not affected by NVAS through 24 d postdosing (80, 90); however, the lung may maintain elevated VA (81).

**TABLE 5:** Characteristics of included studies evaluating vitamin A effects on organ maturation in neonates

Model	Dose amount (*n*/group)	Frequency and age at dosing	Outcomes	Reference
			***Organ weights and hemoglobin***	
Calf	250,000 IU (3)Control (colostrum) (3)	Single, day 1	Hemoglobin and prevalence of anemia not different between groups	(Eaton, 1947) (74)
Human	50,000 IU (809)Control (783)	Single, day 1–3	Hemoglobin and prevalence of anemia not different between groups	(Miller, 2006) (97)
Swine	25,000 IU (16)50,000 IU (16)100,000 IU (16)Control (16)	Single, day 18	Liver weight and relative weight not different between groups	(Surles, 2007) (58)
Swine	25,000 IU (7)50,000 IU (7)100,000 IU (7)Control (7)	Single, day 18	Adrenal, kidney, lung, or spleen weights not different between groups 10-d postdosing	(Sun, 2008) (59)
Swine	6 μg/g (50,000 IU to humans) (4)Control (4)	Multiple, days 5–7	Lung and liver relative weights similar between groups on postnatal day 8	(Ross, 2006a) (79)
Rat	6 μg/g (50,000 IU to humans) (52)Control (52)	Single, day 4	Brain, stomach, intestine, liver, lung, kidney, brown adipose, white adipose, skin, and carcass weights similar through to 24-d postdosing	(Hodges, 2016a; Hodges, 2016b) (80, 90)
Swine	25,000 IU (83)50,000 IU (73)200,000 IU (71)Control (58)	Single, day 1	Liver, lung, kidney, adrenal, and spleen weights similar among group 10-d postdosing	(Gannon, 2017) (76)
			***Organ histology***	
Mouse	6 μg/g (50,000 IU to humans) (6)Control (6)	Multiple, days 1–4	Lung development similar between groups on day 4 of life	(James, 2010) (49)
Rat	6 μg/g (50,000 IU to humans) (4)Control (4)	Multiple, days 5–7	No difference between groups in lung development by tissue density, mean linear intercept, or alveolar development on postnatal day 8	(Ross, 2006a) (79)
Swine	25,000 IU (49–51)50,000 IU (49–51)200,000 IU (49–51)Control (49–51)	Single, day 1	Duodenal or jejunal villus height, crypt depth, or villus:crypt ratio similar among groups between 4- and 10-d postdosing	(Gannon, 2017) (76)

##### Organ maturation

Organ maturation by histological examination was assessed in rats, mice, and swine (Table 5). VA supplementation did not accelerate or attenuate alveolar development (49, 79). Markers investigated included radial alveolar counts, mean linear intercept (airspace volume/surface ratio), and secondary septal crest density. Samples were examined on the final day or day after dosing (day 4 when dosing days 1–4 or day 8 when dosing days 5–7). With limited results on outcomes for organ maturation, the study designs primarily looked for tissue abnormalities (79) or injury from hyperoxia, which was not considered for this review (49). In piglets, NVAS did not affect duodenal or jejunal villus height, crypt depth, or the villus:crypt ratio between 4 and 10 d postdosing (76).

##### Summary

Neonatal VA supplementation showed no effects on hemoglobin, anemia prevalence, organ weight, or lung development, but evidence is limited to short-term evaluation.

### Secondary outcome measures

#### Adverse events

Adverse events or effects were assessed in 4 human studies (72, 98–100), and 1 mouse (49), rat (79), and swine study (76) of those selected for inclusion in the review (**Table 6**). (Note that mortality trials are not included in this systematic review and were summarized in a systematic review and discussed below [34].)

**TABLE 6:** Characteristics of included studies evaluating adverse effects of neonatal vitamin A supplementation

Model	Dose amount (*n*/group)	Frequency and age at dosing	Adverse effect(s) with NVAS	Reference
			***Adverse effects: human studies***	
Human	50,000 IU (112)Control (111)	Single, day 1	Bulging fontanelle, vomiting, loose stools, fever, and irritability not different between groups through 24-h postdosing	(West, 1992) (98)
Human	50,000 IU (1034)Control (1033)	Single, day 1	Bulging fontanelle slightly higher in VA group (4.6 vs. 2.7% at 24 h; 4.5 vs. 2.4% at 48 h)	(Agoestina, 1994) (72)
Human	50,000 IU (398)Control (390)	Single, day 1	Bulging fontanelle, vomiting, irritability, fever, breastfeeding not different between groups at a mean follow-up of 30 h	(Iliff, 1999) (99)
Human	50,000 IU (1032)Control (1013)	Single, day <10	Bulging fontanelle, hospitalization, irritability, fever, frequent stools, and vomiting not different between groups 1–3-d postdosingHospitalization, diarrhea, vomiting, runny nose, and cough not different between groups through 4-wk postdosing	(Nante, 2008) (100)
			***Adverse effects: animal studies***	
Mouse	6 μg/g (50,000 IU to humans) (6)Control (6)	Multiple, days 1–4	No differences in airway epithelial injury, hemorrhage, or macrophage index in mice on postnatal day 4	(James, 2010) (49)
Rat	6 μg/g (50,000 IU to humans) (4)Control (4)	Multiple, day 5–7	No difference between groups in lung injury on postnatal day 8	(Ross, 2006a) (79)
Swine	25,000 IU (7–9)50,000 IU (7–9)200,000 IU (7–9)Control (7–9)	Single, day 1	No substantial effects of treatment among groups for lung, spleen, intestine, or kidney histology. Age related hepatocellular vacuolization was decreased in the control group on postnatal days 7–10	(Gannon, 2017) (76)

VA, vitamin A.

##### Human studies

Human studies looked at bulging fontanelle, intracranial pressure, vomiting, loose stool, fever, irritability, drowsiness, crying, sleeping, and breastfeeding. Acute side effects following dosing were minimal and had similar incidence to control groups. One of the studies found an increase of slight bulging fontanelle in the VA group compared with the placebo group 24 and 48 h postdose (∼4.5 compared with 2.6%) (72), but other studies showed similar incidence between VA and placebo groups. A recent meta-analysis of human NVAS trials with a primary outcome of mortality (including trials excluded for this review) found an increased risk ratio for bulging fontanelle within 48–72 h in NVAS groups compared with placebo groups (1.53; 95% CI: 1.11, 2.11) (34). All other outcomes, including diarrhea, vomiting, fever, feeding, convulsions, crying, jaundice, and various infections did not differ between groups (34).

##### Animal studies

Animal studies looked at lung tissue abnormalities histologically; 1 study found no change in development when assessed for lung injury, tissue density, or mean linear intercept in rats (79), and the other study described no differences in airway epithelial injury, hemorrhage, or macrophage index in mice (49). In piglets, age-related hepatocellular vacuolization was decreased in untreated piglets between 7 and 10 d postdosing, which was likely due to age-related changes in metabolism. No treatment differences were observed in pathological scoring of the lung, kidney, or lymphoid status and extramedullary hematopoiesis in the spleen (76). In the same piglet study, body weight was lower at 10 d in piglets who were administered 25,000, 50,000, and 200,000 IU at birth compared with placebo. This difference in apparent growth rate merits further research to determine optimal weight gain and the mechanism whereby NVAS affects weight gain.

##### Summary

Most evidence supports that NVAS ≤50,000 IU in neonates has no severe acute adverse effects; however, some data indicate that NVAS increases risk of bulging fontanelle.

## Discussion

### Summary of main results

High-dose supplements of VA were absorbed relatively well by neonates, typically mirroring fat absorption and being limited by large fat particle size or prematurity of the neonate. The high doses were taken up primarily by the liver, but also delivered to the lung, kidney, spleen, and adrenal gland. The liver was the primary storage site, and VA concentrations typically showed a dose response to the amount dosed. Extrahepatic tissues did not appreciably store the dose, with organ VA concentrations not showing a dose response and having concentrations similar to, or modestly higher than, controls by 10 d postdosing. Serum retinol concentrations changed dramatically within 1 d postdosing, but either did not rise above 0.7 μmol/L or did not differ from control animals at further time points. VA dosing did not appreciably alter homeostatic mRNA expression or polar metabolite formation in the lung. Supplementation did not affect short-term organ growth or histological analysis.

### Limitations of available research

The studies included were able to offer insight into the absorption, transport, and storage of high-dose VA supplementation to neonates, but limitations existed in outcomes measured. Organ maturation and abnormalities were assessed histologically within 1 d of the last day of dosing either on days 1–4 (49) or 5–7 (79). The authors mention that they were assessing short-term uptake and therefore more long-term outcomes were not assessed; differences in development were not expected. Increased lung alveolar septation in rats injected with retinoic acid daily from days 3 to 13 was observed on day 14 (101). The present review sought to examine oral doses of VA, not injected doses or doses given as retinoic acid. Whether NVAS positively or negatively affects organ development over time at the cellular level remains unknown.

Substantial decreases observed in lung and other organ VA from 1 to 5 d postdose indicate rapid utilization or metabolism of the VA dose (76, 80, 89). Furthermore, α-retinol doses transported to measured extrahepatic organs (i.e. kidney, lung, spleen, and adrenal gland) returned to negligible concentrations by 7 d postdose (93), indicating rapid utilization, metabolism, or recycling back to the liver on lipoproteins of α-retinol. Although these data indicate that retinoid concentrations decrease relatively soon after high-dose supplementation, it is unknown by which mechanism this occurs.

VA (retinol) is oxidized in vivo to form retinal and ultimately retinoic acid, the hormone form of VA that is thought to support most of the effects of VA outside of the visual cycle (45, 102). However, retinoic acid concentrations, notably in serum, are tightly regulated at very low concentrations (i.e. 1–3 nM), much lower than VA (1–4 μM), making their detection and study difficult (102). Furthermore, retinol and retinoic acid can both be metabolized to glucuronides, hydroxy-, oxo-, and other metabolites following supplementation (103), and are found at similarly low concentrations as retinoic acid (102). Although it was historically considered that these metabolites are primarily degradation products of retinol and retinoic acid, studies have shown biological activity of retinoyl β-glucuronide (104), indicating that VA metabolism is not simply utilization or degradation. Aside from pooled aqueous radioactivity in lungs after a [^3^H]-labeled dose (79), these metabolites were not analyzed after dosing in a neonatal period. Assessing tissue specificity and flux through the retinoic acid pathway and to other metabolites could give a more detailed understanding of how the VA dose is metabolized and what effects it has.

### Public health implications and future directions

The WHO does not currently recommend NVAS as a public health intervention to reduce infant morbidity and mortality. There is consensus that there is variation in effect estimates of NVAS; however, the source and level of this heterogeneity remains a topic of interest and discussion.

A commentary by Haider and Bhutta noted no overwhelming or consistent evidence for NVAS, and that VA status likely varies by study site and is dependent on maternal status (33). They suggest that there is probably no single magic bullet to improve VA status in populations at risk of deficiency, and suggest focusing on strategies to improve maternal nutrition including dietary diversification, family planning, and micronutrient supplementation.

Follow-up commentaries have provided additional perspective, interpretation, and suggested future implementation or research directions regarding NVAS. West and Sommer suggest that clear regional difference of NVAS's impact on mortality between Africa and Asia, which is likely influenced by baseline VA status, merits regional-specific implementation of NVAS (105). Benn, Aaby, and Fisker hypothesize that NVAS leads to negative mortality outcomes for females once they start receiving the DTP vaccine, and propose to analyze data for these potential interactions (106). Fisker, Aaby, and Benn identified links between NVAS and C-reactive protein concentrations, suggesting NVAS has effects on the immune system and should be further researched (107). Boucher suggests that vitamin D is an important covariate to keep in mind when considering NVAS and VA metabolism due to their interactions and should also be analyzed further (108).

Data covered in this review suggest that NVAS only transiently increases extrahepatic tissue VA; however, the extent of potential longer terms effects, such as immunomodulation or immune memory, require further study. Liver stores are increased longer than other tissues, but it is not clear for how long, and whether this VA is reaching target tissues. Numerous tracer studies have indicated that a substantial amount of recently ingested VA is delivered to target tissues on chylomicra. Approaches to deliver VA more consistently may be necessary to maintain a steady flux of VA to the rapidly developing extrahepatic organs. These approaches include more frequent supplementation, ensuring breastfeeding or nutritionally adequate substitutes, and improving the quality and composition of breast milk through fortification, biofortification, and dietary diversity. Care must be taken because overlapping VA interventions can lead to high intakes and status.

When aiming to optimize the status of a particular nutrient in an individual or population, it is critical to have adequate nutritional assessment methodology and implementation to: *1*) identify populations and subgroups who have inadequate status, and *2*) evaluate and monitor interventions to ensure that they are both safe and efficacious. Numerous researchers have called for broadening the approach to expand knowledge regarding NVAS, nutrient-nutrient interactions, and immunomodulation. Suggested efforts to ensure neonatal VA status include improving maternal nutritional status through more frequent intake of VA-containing foods, promoting proper feeding for infants and young children, and reducing the burden of nutrient-depleting infections.

## Supplementary Material

nmaa137_Supplemental_Files

## Notes

WHO acknowledges the Bill & Melinda Gates Foundation (OPPHJ5297) for their financial support to the WHO Department of Nutrition and Food Safety and the International Life Sciences Institute that supplied travel funds to BMG to assist with the conduct of this systematic review in Geneva, Switzerland.

Supplemental Figures 1 and 2 are available from the “Supplementary data” link in the online posting of the article and from the same link in the online table of contents at https://academic.oup.com/advances.

Abbreviations used: DTP, diphtheria, tetanus, and pertussis; GIT, gastrointestinal tract; LMIC, low- and middle-income country; MRDR, modified relative dose response; NVAS, neonatal vitamin A supplementation; RAE, retinol activity equivalents; RBP, retinol-binding protein; VA, vitamin A.
