# Supplementary material for: Metabolism of Neonatal Vitamin A Supplementation: A Systematic Review
Source: Adv Nutr. 2020 Nov 19;12(3):942–58. doi: 10.1093/advances/nmaa137 (PMC8262574; doi:10.1093/advances/nmaa137)
Supplement: nmaa137_Supplemental_Files [file nmaa137_Supplemental_Files.zip › Supplementary Figure 1 (data extraction)'.pdf]

Systematic review of the metabolism of high dose vitamin A supplementation in the neonatal period
Gannon "Online Supplementary Material"

Supplementary Figure 1 – **Data extraction outline**

**Trial methods**
• Study design<br>• Unit and method of allocation<br>• Masking of participants and outcome assessors<br>• Exclusion of participants after randomization and proportion of losses to follow-up
**Participants**
• Location of the study<br>• Sample size<br>• Age at dosing and follow-up<br>• Sex<br>• Gestational age<br>• Birth weight<br>• Breastfeeding practices<br>• Vitamin A status of mothers<br>• Vitamin A supplementation of mothers<br>• Inclusion and exclusion criteria
**Intervention**
• Dose of vitamin A<br>• Form of vitamin A<br>• Timing of supplementation in hours or days<br>• Supplementation regimen (*e.g.* single, multiple doses)<br>• Co-intervention
**Comparison**
• Type of comparison (*e.g.* no intervention, placebo or same supplement without vitamin A)
**Outcomes**
• Primary and secondary outcomes

Outline of the data extraction form. For each study that met the inclusion criteria, data relevant to each category were recorded electronically, synthesized, and reported in the results section. While data analysis by subgroup was initially planned, limited data prevented subgroup analysis, and data were presented narratively instead.
