# Supplementary material for: Metabolism of Neonatal Vitamin A Supplementation: A Systematic Review
Source: Adv Nutr. 2020 Nov 19;12(3):942–58. doi: 10.1093/advances/nmaa137 (PMC8262574; doi:10.1093/advances/nmaa137)
Supplement: nmaa137_Supplemental_Files [file nmaa137_Supplemental_Files.zip › Supplementary Figure 2 (PRISMA diagram).pdf]

Supplementary Figure 2 – **PRISMA diagram**

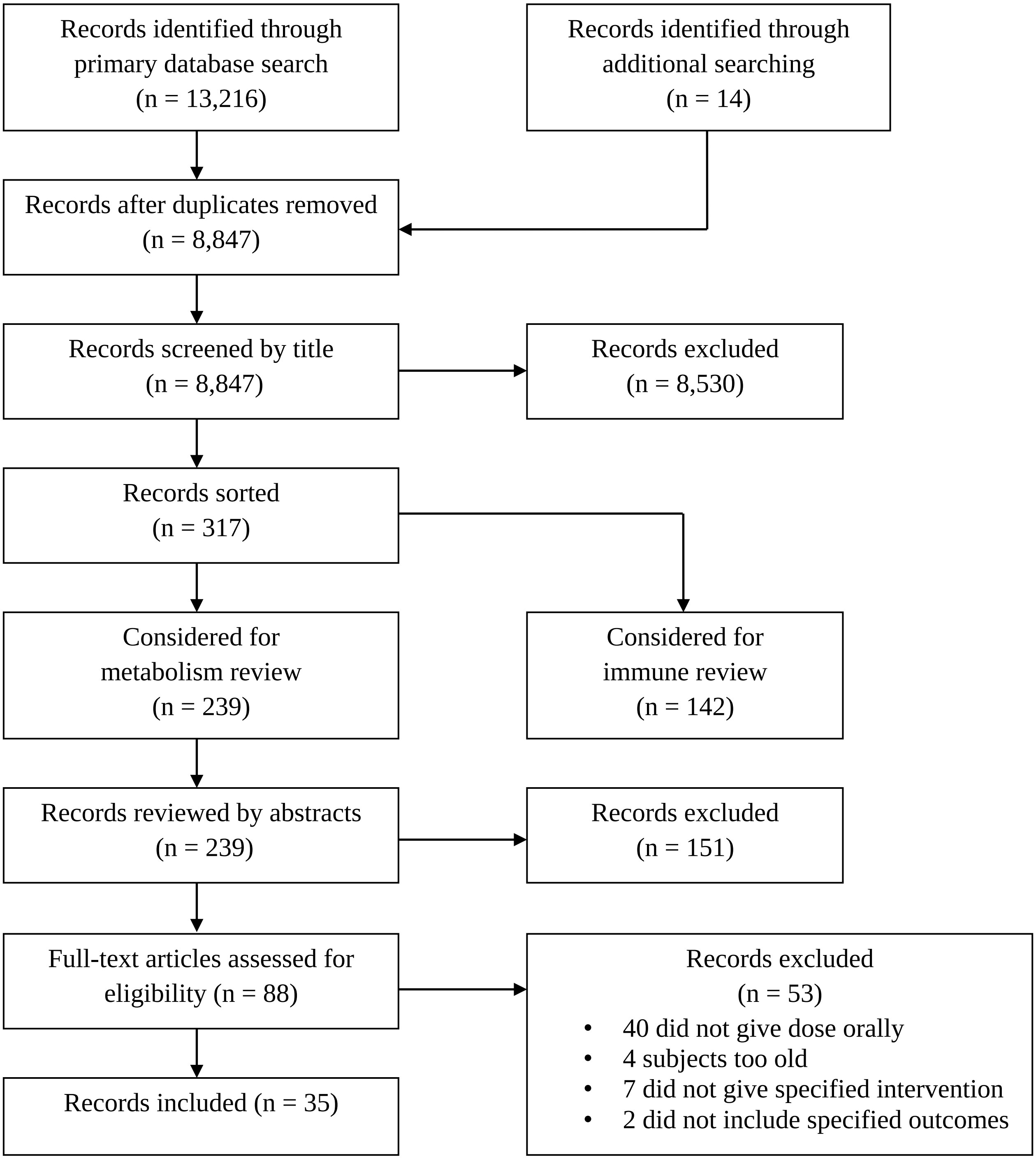

PRISMA flow diagram of records from those identified in the search to those ultimately selected for inclusion in the review. Reasons for exclusions when full-text articles were assessed are noted. Only the number excluded is reported for records that were excluded solely based on the title or abstract.
